# Proton Therapy for Primary Bone Malignancy of the Pelvic and Lumbar Region – Data From the Prospective Registries ProReg and KiProReg

**DOI:** 10.3389/fonc.2022.805051

**Published:** 2022-02-16

**Authors:** Rasin Worawongsakul, Theresa Steinmeier, Yi-Lan Lin, Sebastian Bauer, Jendrik Hardes, Stefanie Hecker-Nolting, Uta Dirksen, Beate Timmermann

**Affiliations:** ^1^ Department of Particle Therapy, University Hospital Essen, West German Proton Therapy Centre Essen, Essen, Germany; ^2^ Radiation Oncology Unit, Department of Diagnostic and Therapeutic Radiology, Ramathibodi Hospital, Mahidol University, Bangkok, Thailand; ^3^ West German Cancer Centre Network, Essen, Germany; ^4^ Department of Medical Oncology, Sarcoma Center, West German Cancer Center, University of Duisburg-Essen, Essen, Germany; ^5^ German Cancer Consortium (DKTK), Essen, Germany; ^6^ Department of Orthopedic Oncology, University Hospital Essen, Essen, Germany; ^7^ Pediatrics 5 (Oncology, Hematology, Immunology), Klinikum Stuttgart Olgahospital, Stuttgart, Germany; ^8^ Pediatrics III (Hematology, Oncology, Immunology, Cardiology, Pulmonology), University Hospital Essen, Essen, Germany

**Keywords:** Proton therapy, bone malignancy, bone tumor, sarcoma, pelvic, lumbar

## Abstract

**Purpose/Objective(s):**

Multimodality treatments together with local proton therapy (PT) are commonly used in unresectable primary bone malignancies in order to provide better tumor control rate while maintaining good feasibility. The aim of this study is to provide data on outcome of PT for the challenging cohort of pelvic and lumbar bone tumors.

**Methods and Materials:**

This retrospective study includes all patients with primary bone malignancy of the pelvis and lumbar spine receiving PT in our institution between May 2013 and December 2019 enrolled in the prospective registries KiProReg and ProReg collecting information on demographics, treatment, tumor characteristics, toxicities, and outcome.

**Results:**

Eighty-one patients were enrolled with a median age of 19.7 years (1.3–85.8). The median follow-up time was 27.5 months (1.2–83.2). The majority of patients was male (64.2%), ECOG status of 0–1 (75.2%), underwent only biopsy (50.6%), received chemotherapy (69.1%) and was assigned for definite PT (70.4%). The predominant tumor characteristics were as follows: Ewing’s sarcoma histology (58%), negative nodal involvement (97.5%) and no metastasis at diagnosis (81.5%). Median maximal diameter of tumor was 8 cm (1.4–20). LC, EFS and OS rate were 76.5, 60, and 88.1% at two years and 72.9, 45.7, and 68.9% at three years, respectively. Age over 20 years was a significant negative factor for LC, EFS, and OS. Metastatic disease at initial diagnosis affected OS and ECOG status of 2–4 affected EFS only. Regarding 17 relapsed cases (21%), isolated distant relapse was the most common failure (46.9%) followed by local failure (40.6%). Eleven out of 14 evaluable patients relapsed within high-dose region of radiotherapy. Acute grade 3–4 toxicity was found in 41 patients (50.6%) and all toxicities were manageable. Late grade 3 toxicity was reported in 7 patients (10.4%) without any of grade 4. Most common higher grade acute and late side effects concerned hematologic and musculoskeletal toxicity.

**Conclusion:**

Proton therapy resulted in good oncological outcomes when being part of the multimodality treatment for pelvic and lumbar primary bone malignancies. However, distant metastases and local failures within the high-dose region of radiotherapy are still a common issue. Acute and late toxicities of combined therapy were acceptable.

## Introduction

Primary bone malignancy is a rare malignant disease (1, 2). Resection is still the main curative local treatment for bone tumors (3), but not all patients are suitable for total tumor removal with adequate margins, especially for tumors of difficult locations as pelvis and lumbar spine (4, 5). Due to the close proximity to important normal structures, complete surgery of the tumor in these regions can cause unacceptable morbidity to patients. However, worse survival rates have been reported in patients not having total resection (3, 5, 6). Thus, radiotherapy will play a major role in these patients to improve local control and survival rates. However, high doses of radiotherapy are needed due to the radioresistant nature of bone tumor potentially leading to relevant toxicity (7–10). One way to minimize this risk for treatment complication is the use of proton therapy (PT). While proton passes through the body of a patient, it releases kinetic energy in the certain depth without any dose exposure to normal tissue distal to this area. The peak of kinetic energy deposited in tissue is called Bragg peak. Due to this physical advantage, PT offers the chance to increasing RT doses while lowering the burden to the surrounding normal tissues (11, 12).

While clinical data on proton therapy in primary bone malignancy of the pelvic and lumbar area is still limited, this study provides clinical tumor outcome, toxicity and pattern of failure after treatment with proton therapy from our prospective registries embedded in a large interdisciplinary sarcoma center and the national study framework.

## Materials and Methods

### Patient Selection

Patients from both the prospective ProReg (Registry number: DRKS00004384) and KiProReg registries (Registry number: DRKS00005363) with primary diagnosis of Ewing’s sarcoma, chondrosarcoma, chordoma, osteosarcoma, and osteoblastoma with tumor locations of the pelvis and lumbar spine who started proton treatment in our institution between May 1, 2013 and December 31, 2019 were included in this analysis. Approval of the local ethics committee for ProReg (12-5143-BO) and for KiProReg (13-5544-BO) had been obtained. All patients had signed informed consent for enrollment into the respective registry. Database covered data collection on demographics, treatment, tumor characteristics, survival and toxicities.

### General Treatment Approach

All the files of the patients were discussed in a multidisciplinary tumor board with regard to the appropriate treatment decision including surgical approach for each patient before starting PT. The treatment was applied according to the recommendation from the tumor board and European treatment protocols such as the EURO-EWING for Ewing’s sarcoma and the EURAMOS for osteosarcoma, respectively. In addition, patients with chordoma and chondrosarcoma were treated according to in-house standard of practice (SOP) (13). In these protocols, wide or marginal resection was recommended for patients if the risk of surgery was manageable and acceptable post-operative morbidity was expected. For histologies such as Ewing’s sarcoma, high-graded chondrosarcoma and osteosarcoma, chemotherapy was given to patients according to the protocol. If gross total tumor resection with oncologically appropriate surgical margins could not be achieved or poor response to chemotherapy was reported, radiotherapy was introduced. Therefore, adjuvant radiotherapy was still considered for chordoma and chondrosarcoma patients who achieved gross total (R0 or R1) resection. The radiation dose and volume depended on the extent of resection, histopathology and timing of radiotherapy.

### Radiotherapy Concept

Patients were set-up either in supine or prone position depending on dorsal or ventral location of tumor. Immobilization was assured with individually customized vacuum casts. In pelvic tumors, bladder filling protocol, either *via* drinking or suprapubic cystostomy (the latter particularly for pediatric patients), were considered case by case in accordance with the location of tumor. All patients with lumbar tumors were planned for treatment with empty bladder. After performing planning CT with internal or external planning MRI, import and matching of diagnostic MRI at first diagnosis and during course of treatment to the Raysearch (RaySearch Laboratories, Stockholm, Sweden) planning system were done for target volume delineation. GTV1 was contoured according to the initial tumor volume and defined as the tumor bed after adaptation to any geometrical changes. Gross residual tumor at the time of PT was contoured as GTV2. CTV1 was generated from tumor bed plus a CTV margin depending on histology: 1.5–2 cm for Ewing’s sarcoma, 1 cm for chordoma and chondrosarcoma, 2 cm for osteosarcoma and 0.5–2 cm for osteoblastoma. The next level of prescription dose was defined differently according to histology. Whereas CTV2 of chordoma, chondrosarcoma and osteosarcoma was GTV2 (CTV2 = GTV2), CTV2 of Ewing’s sarcoma was tumor bed (CTV2 = Tumor bed). No CTV2 was defined for osteoblastoma. Subsequently, additional boost to GTV2 as CTV3 was considered for Ewing’s sarcoma patients with gross residual disease (see **Supplementary 1**). Safety PTV margin of 5 mm was used in the pelvic and lumbar location of tumor.

The dose of proton therapy was calculated taking into account the RBE expressed in Gy (RBE), which equals the absorbed dose in Gray of protons multiplied by 1.1. Thus, dose constraints for plan evaluation were all determined in Gy (RBE). Proton treatment can be planned either sequentially boost, which we used homogenous dose and reduced volume for the boost in the later phase, or simultaneous integrated boost (SIB), which we boost to high-risk region simultaneously by using heterogenous dose distribution. Equivalent biological dose was calculated for SIB planning to provide same radiobiological effect as sequential treatment. Dose to organ at risks (OARs) was determined as a maximal dose of 50 Gy (RBE) to spinal cord, maximal dose of 66 Gy (RBE) and mean dose of 55 Gy (RBE) to caudal sac, mean dose 45 Gy (RBE) to femoral heads in adults and 26 Gy (RBE) in children, mean dose of 50 Gy (RBE) to penile bulb and mean dose of testis of 3 Gy. Volume of bowel exposure to a dose of 45 Gy (RBE) or more should be kept below 195 ml. Mean dose of kidneys should be kept below 18 Gy and volume exposure to a radiation dose of 15 Gy or higher should be kept below 65% for one side or below 20–25% for both sides. Volume of bladder and rectum receiving doses of more than 50 Gy should be kept below 60% and 50%, respectively. Whole involved vertebras were covered with at least 20–30 Gy in case of pre-puberty patients to stop bone growth symmetrically and reduce scoliosis in the future.

### Staging and Follow-up

For all patients, relevant staging information was requested before treatment. Information included initial, postoperative and recent MRI imaging, surgery reports, general medical report, neurological status, blood count, lung and bone screening and additional investigations if appropriate like rectoscopy, or for bladder and kidney function. All of the patients had clinical base-line evaluation before starting PT. Weekly clinical assessments were performed in all patients during proton therapy. Blood count was requested and performed on a regular basis if chemotherapy was applied or if the field of PT was considered to potentially affect bone marrow. After completion of treatment, all patients should have personal appointment at the institution at 90 days after PT and then yearly on basis. If they were not able or willing to have their check-up in person, written inquiries by questionnaires and telephone interviews were performed. At the same time points, all relevant medical documents and imaging including reports were requested. During COVID-19 pandemic, appointments by telephone without personal visits were suggested.

### Pattern of Failure Evaluation

Pattern failure was also reported within the registries. In case of local recurrence, the MRI at time of progression was imported and fused with planning CT in Raysearch planning system. Local failure pattern was scored according to Dawson et al. (14). High-dose region recurrence was defined if local tumor progression of more than 20% in size or relapse was situated with more than 95% volume inside 95% of total cumulative prescription dose. Lower-dose region recurrence was defined if more than 95% volume of relapsed tumor was covered by 95% of the prescription dose for CTV1. Local failure was considered “marginal” if 20 to 95% of recurrent tumor volume was within 95% of the dose to CTV1. If image data at time of progression was not available, it was specifically requested. If finally only report was available, local recurrence was registered but not scored according to type of local recurrence.

### Toxicity Evaluation

All toxicities were assessed and graded prospectively according to the Common Terminology Criteria for Adverse Events (CTCAE) version 4.0. All patients were evaluated before the start of PT, weekly during PT and then as explained above. Higher-grade toxicities were defined as CTCAE grade 3 or higher. Whereas acute toxicities were defined as any adverse events occurring during PT and before 3 months after completion of PT, late toxicities were defined as any adverse event occurring since 3 months after completion of PT.

### Statistics

This study analyzed data retrospectively. Qualitative data were presented as frequency and percentage, quantitative data was reported as median and range. Follow-up time was calculated from first diagnosis to last contact of patient or death. Overall survival (OS—time from diagnosis to dead of any cause) was the primary objective. Both local control (LC—time from diagnosis to local recurrence or progression) and event-free survival (EFS—time from diagnosis to any event) were the secondary objectives for this study. They were all analyzed with the Kaplan–Meier Method. Any recurrence or death of any cause was defined as an event. After univariate analysis with log-rank test, multivariate analysis for factors which have or tend to have effect on local control, EFS and OS was conducted with Cox regression test, respectively.

All statistical analyses were performed in 95% confidence interval (5% alpha risk) using IBM SPSS Statistics version 27.

## Results

Eighty-one patients were eligible for this study. Characteristics of patients are displayed in **Table 1**. Median and mean age were 19.7 and 30.5 years (1.3–85.8 years). The majority of patients were male (64.2%) with good ECOG performance status of 0-1 (71.6%) treated with curative intent (97.5%). The most common histopathology was Ewing’s sarcoma family tumor (58%), followed by chordoma (24.7%), chondrosarcoma (7.4%), osteosarcoma (7.4%) and osteoblastoma (2.5%). Tumors were located either in pelvic and sacral sites (84%) or the lumbar region (16%). The median tumor size was 8 cm (1.4 – 20 cm). The majority of patients did not have any nodal involvement (97.5%) or other metastatic disease at time of diagnosis (81.5%). Nodal involvement was found in only 2 patients and all of them were diagnosed with Ewing’s sarcoma.

**Table 1:** Patient Demographics, Tumor and Treatment Characteristics.

	N (%)
Number of patients (%)	81 (100)
Mean age at diagnosis (years)	30.5
Median age at diagnosis (years)	19.7
(range)	(1.3–85.8)
≤20 years (n)	42 (51.9)
>20 years (n)	39 (48.1)
Sex	
Female	29 (35.8)
Male	52 (64.2)
ECOG performance status	
0–1	58 (71.6)
2	9 (11.1)
3	11 (13.6)
4	1 (1.2)
Unknown	2 (2.5)
Location of tumor	
Pelvis and sacrum	68 (84)
Lumbar	13 (16)
Histology	
Ewing’s sarcoma	47 (58)
Chordoma	20 (24.7)
Chondrosarcoma	6 (7.4)
Osteosarcoma	6 (7.4)
Osteoblastoma	2 (2.5)
Tumor size (median in cm)	8.00
(range)	(1.4–20)
N staging	
N0	79 (97.5)
N+	2 (2.5)
M staging	
M0	66 (81.5)
M+, Lung only	7 (8.6)
M+, Non-Lung	2 (2.5)
Combined	6 (7.4)
Surgery	
Biopsy only	41 (50.6)
R0 resection	12 (14.8)
R1 Resection	3 (3.7)
R2 Resection	19 (23.5)
Rx resection	6 (7.4)
Chemotherapy	
No	25 (30.9)
Any chemotherapy	56 (69.1)
Any concurrent	43 (53.1)
Any before PT	55 (67.9)
Any after PT	45 (55.6)
PT Timing	
First RT course	79 (97.5)
Re-irradiation	2 (2.5)
Location of PT	
Primary tumor	70 (86.4)
Recurrence tumor	7 (8.6)
Primary and metastatic sites	4 (5)
Indication of PT	
Definite	57 (70.4)
Adjuvant	18 (22.2)
Pre-operative	6 (7.4)
Median radiation dose	59.4
(range)	(45–74)
Definite	59.4 (50.4–74)
Adjuvant	55 (45–74)
Pre-operative	50.4 (45–54)
Technique of PT	
Sequential	66 (81.5)
SIB	15 (18.5)
Median interval (range):	
From surgery to PT	147.5 (34–519)
From PT to Surgery	69.5 (20–153)

PT, Proton therapy; SIB, Simultaneous integrated boost.

With regard to the surgical approach, the majority of patients underwent only biopsy (50.6%), in 14.8% R0-resection was achieved, whereas in 3.7% R1-resection and in 23.5% R2-resection was confirmed, respectively. Six patients who underwent total resection, resection status could not be categorized either R0 or R1 (Rx). In the present cohort, more than half of the patients received concurrent chemotherapy (53.1%) and even more (69.1%) received some chemotherapy at any time. Regarding all patients in this cohort, 67.9% had chemotherapy before receiving PT and 55.6% received chemotherapy following PT. Despite neoadjuvant chemotherapy, only 12 patients received definite surgery. In eleven of them pathological reports were available. While seven patients of them showed good response (more than 90% necrosis of tumor), in four patients response to chemotherapy was poor. Whereas all patients diagnosed with Ewing’s sarcoma and osteosarcoma were treated with chemotherapy according to the respective protocol, none of the chordoma patients or any osteoblastoma patients received any chemotherapy. In chondrosarcoma, however, half of the patients who had high-graded chondrosarcoma with a higher risk for distant metastasis received chemotherapy as part of the multimodality treatment.

With regard to radiotherapy, most patients had 1st course radiotherapy treatment (97.5%) with curative intent. Approximately 86.4% of patients received radiotherapy only at primary lesion for the first course of treatment at initial diagnosis, 5% at both primary and metastatic sites and 8.6% at primary location after recurrence. In 70.4% of the cohort, radiotherapy was given as definite local therapy. Postoperative radiotherapy was given in 22.2% and pre-operative radiotherapy in 7.4% of the patients, respectively. Prescription doses differed according to histopathology, extent of resection and timing of radiotherapy. Median radiotherapy dose of 59.4 Gy in Ewing’s sarcoma, 74 Gy in chordoma, 69.3 Gy in chondrosarcoma, 70 Gy in osteosarcoma and 54 Gy in osteoblastoma were applied (see **Supplementary 2**). Sixty-six patients (81.5%) were treated with sequential cone-down technique and 15 patients (18.5%) were treated with SIB technique (**Table 1**).

Median time from surgery to radiotherapy was 147.5 days (34–519) for post-operative treatment, and median time from radiotherapy to surgery was 69.5 days (20–153) for pre-operative radiotherapy, respectively.

### Survival

**Figure 1:**
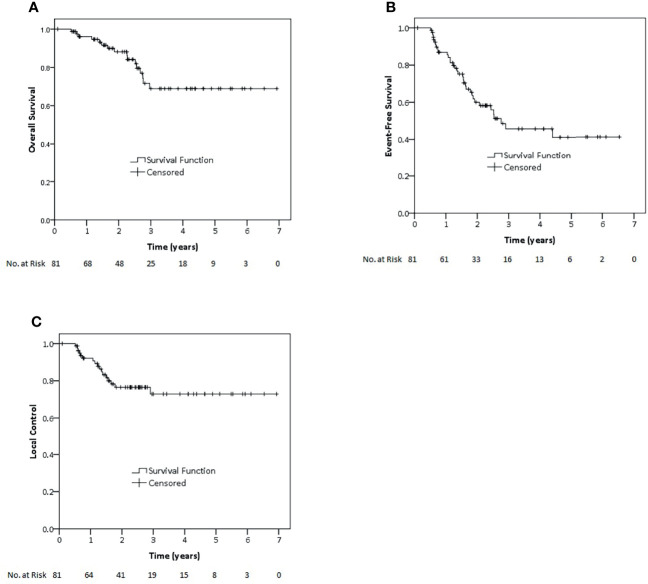
Kaplan–Meier estimates of overall survival **(A)**, event-free survival **(B)** and local control **(C)** rates for all patients of this study.

Median follow-up time for all 81 patients in this cohort was 27.5 months (12–83.2 months). Two-year and 3-year OS for all patients in this cohort were 88.1 and 68.9%, respectively. While 2-year and 3-year EFS were 60 and 45.7%, 2-year and 3-year LC were 76.5 and 72.9%, respectively (**Figure 1**). Regarding non-metastatic patients, 2-year and 3-year OS were 89.1 and 77.4%. EFS rates at 2 and 3 years were 60.2 and 48.5%, whereas LC rates at 2 and 3 years were 72.9 and 68.8%, respectively.

**Figure 2:**
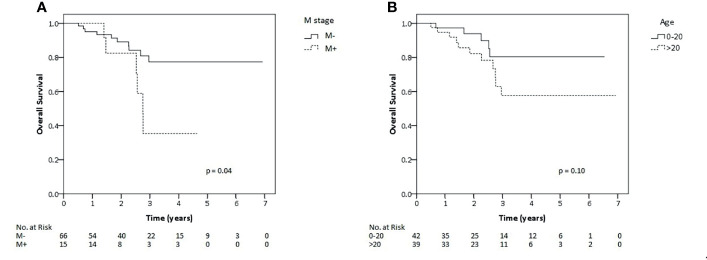
Kaplan–Meier estimates of overall survival rates for all patients of this study according to metastatic status **(A)** and age **(B)**.

Within univariate analysis for the whole cohort (**Supplementary 3**), age older than 20 years displayed an impact on LC and EFS and metastatic disease impacted on OS (**Figure 2**). Furthermore, ECOG performance status of 0–1, tumor of 10 cm in size or lower and any resection of tumor showed borderline significance with regard to superior EFS. Nodal metastases tended to have impact on survival. Accordingly, those factors were analyzed also within multivariate analysis except for nodal metastasis due to very low number of patients on nodal positive arm (n = 2). Within the multivariate testing, age older than 20 years had a significant detrimental effect on LC (HR 8.77, p <0.01), EFS (HR 4.37, p <0.01) and OS (HR 4.8, p = 0.02), respectively. Furthermore, metastatic patients had significantly inferior OS (HR 3.72 p = 0.02) and worse performance ECOG status, scored 2–4, had significantly poorer EFS (HR 2.38, p = 0.04 (**Table 2**). However, we also analyzed correlation between local relapse and surgical status. Whereas, 11 of 41 (26.8%) patients who underwent only biopsy developed local relapse, six of forty patients who underwent any surgery (15%) also experienced local relapse. Anyway, no statistical significant difference was observed between both groups and even in patients who achieved R0-resection, two of twelve patients (16.7%) still experienced local relapse.

**Table 2:** Multivariate analysis of all patients.

Factors	Local control		EFS		OS	
	HR (95% CI)	p-value	HR (95% CI)	p-value	HR (95% CI)	p-value
Age	**8.77 (1.99–38.66)**	**<0.01**	**4.37 (1.95–9.8)**	**<0.01**	**4.8 (1.31–17.6)**	**0.02**
≤20 vs >20						
Performance status	2.16 (0.7–6.72)	0.18	**2.38 (1.06–5.34)**	**0.04**	2.26 (0.68–7.55)	0.19
0–1 vs 2–4						
Tumor size	1.18 (0.37–3.78)	0.78	1.54 (0.67–3.53)	0.31	1.91 (0.55–6.59)	0.31
≤10 vs >10 cm						
M staging	0.35 (0.04–2.93)	0.33	1.4 (0.53–3.69)	0.5	**3.72 (1.21–11.44)**	**0.02**
M0 vs M1						
Surgery	0.82 (0.28–2.4)	0.71	0.65 (0.31–1.37)	0.25	0.88 (0.28–2.8)	0.83
No resection vs any resection						

**Figure 3:**
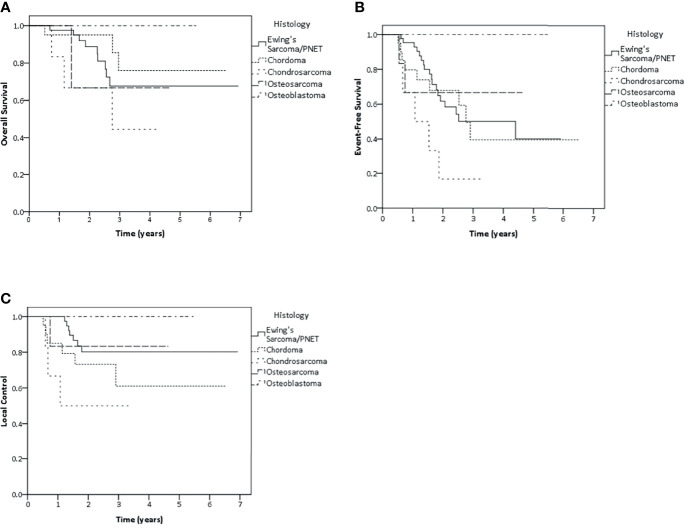
Kaplan–Meier estimates of overall survival **(A)**, event-free survival **(B)** and local control **(C)** rates according to histology.

Ewing’s sarcoma patients represented the largest subgroup in this cohort (n = 47 patients) and were analyzed separately. The results of both 2-year and 3-year LC were 80.2%. Whereas, 2- and 3-year EFS were 61.7 and 50.1%, OS rate at 2 and 3 years were 88.7 and 67.7%, respectively (**Figure 3**). Tumor larger than 10 cm showed negative impact on LC and EFS. While, patient older than 20 years showed poorer EFS rate and patient with nodal metastasis had impact on survival, radiotherapy at primary as initial treatment showed better EFS and OS than radiation after recurrence (**Supplementary 4**). Nodal metastasis was not included for the further multivariate analysis due to the same reason as mentioned before. As a result of multivariate analysis, only significant adverse effect on EFS was found for older age (>20 years of age) (HR 4.51, p <0.01), Tumor of more than 10 cm (HR 4.16, p = 0.02) and radiation for recurrence versus initial therapy (HR 7.59, p <0.01). However, none of the factors showed any significant effect on LC or OS (**Supplementary 5**).

Concerning other histology, 2-year and 3-year LC rate were 73.2 and 61% in chordoma group. Whereas both 2-year and 3-year LC rate were 50% in chondrosarcoma patients, 83.3% LC rate in osteosarcoma patients and 100% LC rate in osteoblastoma patients were found in both 2 and 3 years (**Figure 3**).

### Pattern of Failure

During follow-up, 32 patients experienced progressive disease. The most common cause of progression was distant failure in 15 patients (46.9%), followed by local failure in 13 patients (40.6%) and combined local and distant failure in 4 patients (12.5%), respectively.

Median time to local relapse was 14.7 months (6.2–34.9). Out of the 17 patients experiencing local relapse with or without distant metastasis, in 14 all required information for matching of relapse imaging data with RT plan was available. Eleven (64.7%) had progression or relapse in the high-dose region of PT. Two patients had local failure within the lower dose region and 1 patient had marginal failure of tumor. The later patient unfortunately experienced synchronous extensive metastatic failure, too.

### Toxicity

During the course of PT, overall higher-grade acute toxicity was documented in 41 patients (**Table 3**). The most common acute higher-grade toxicity was hematologic toxicity, occurring in 34 patients out of 72 patients having blood counts on a regularly basis. In 15 patients, higher-grade gastrointestinal toxicity, and in 7 higher-grade skin toxicity occurred. Regarding hematologic toxicity, all concerned 34 patients with higher-grade toxicity received chemotherapy before or concurrent to proton treatment. The most common type of hematologic toxicity was leukopenia occurring as grade 3 or 4 in 33 patients. One patient presented with ileostomy before the beginning of the radiotherapy session, which was defined as non-radiotherapy-related grade 4 gastrointestinal toxicity. Three patients needed unplanned hospitalization for reasons other than receiving chemotherapy (due to febrile neutropenia, accident, and hematoma of left knee of unknown cause, respectively). Five patients had more than three days of treatment interruption, either due to toxicity or to machine issues.

**Table 3:** Acute toxicity report for all patients.

System	N	Grading of toxicity (N)				
		Grade 0	Grade 1	Grade 2	Grade 3	Grade 4
**General**	81	15	40	24	2	0
**Skin**	81	2	22	50	7	0
**GI**	81	41	23	12	4	1
**GU**	79	52	14	11	2	0
**Musculoskeletal**	81	60	17	2	2	0
**Psychology**	31	27	4	0	0	0
**Neuro**	31	18	10	3	0	0
**Hematology**	72	14	11	13	18	16
**Overall**	81	0	8	32	24	17

GI, gastro-intestinal; GU, Genito-urinary.

After radiotherapy, data on late toxicity (**Table 4**) during follow-up was available for 67 patients (82.7%) so far. Higher-grade toxicities were found in seven out of 67 patients (10.4%). The most common toxicity was found regarding the musculoskeletal system for three patients (bone pain in 2, bone deformity in 1).

**Table 4:** Late toxicity report for all patients.

System	N (%)	Grading of toxicity (N)				
		Grade 0	Grade 1	Grade 2	Grade 3	Grade 4
**General**	65 (80.2)	31	16	16	2	0
**Skin**	64 (79)	26	25	13	0	0
**GI**	65 (80.2)	50	9	4	2	0
**GU**	62 (76.5)	45	10	6	1	0
**Musculoskeletal**	66 (81.5)	44	10	9	3	0
**Psychology**	47 (58)	38	5	4	0	0
**Neuro**	48 (59.3)	41	5	2	1	0
**Hematology**	0	0	0	0	0	0
**Overall**	67 (82.7)	12	19	29	7	0

GI, gastro-intestinal;

GU, Genito-urinary.

## Discussion

Due to the rarity of primary malignant bone tumors, data on RT are sparse, particularly with regard to modern RT technologies. This study was performed in retrospective manner to provide clinical data of 81 patients treated with modern PT, focusing on primary bone malignancy located in the pelvic and lumbar region. In general, RT for bone tumors is challenging due to the need for high doses at delicate sites. Principally, PT can provide some benefits due to the advantage of physical characteristics in order to spare normal tissue. However, clinical data for proton therapy treatment is still limited today.

When compared to other studies, our patient cohort seems to display unfavorable risk factors. All patients included in this analysis had tumors located in the pelvic and lumbar region, which is known to have worse prognosis when compared to those in the extremities (15–18). Furthermore, majority of our patients had large tumor size which also understood as a negative predicting factor (5, 15) and complete tumor resection was not considered feasible. Some patients also had nodal or distant metastatic disease at diagnosis which considered as high-risk in sarcoma patients. So, chemotherapy and relatively high doses of radiation for treatment were required in majority of patients in this cohort.

Still, the results of the survival analysis were satisfactory in this study. Overall, the results of our study were similar or even somewhat superior when compared to other studies despite the particularly unfavorable cohort we had investigated. Systematic review outcomes of primary pelvic bone sarcoma from 2018 showed 5-year LC of 81.7% and 5-year OS of 55% (19). In addition, Kerr, et al. (2019) studied the treatment outcomes of primary spinal bone malignant tumor and reported 5-year OS in different histological types of primary bone tumor. The best prognosis was observed in chordoma patients with 5-year OS of 70%, followed by chondrosarcoma with 5-year OS of 69% and Ewing’s sarcoma with 5-year OS of 62%. The most unfavorable outcome was reported for osteosarcoma, with only 38% survival rates at 5 years (5). When comparing our study with some surgical series, the results are still very much alike. Laitinen et al. reported 5-year LC and 5-year disease-specific survival (DSS) of 58 and 70.2%, respectively, and 10-year DSS of 62.9% (6). Another surgical series from 2016 reported on patients very high local control and survival at 5 years of 91.3 and 84.4%, respectively (20). However, this surgical series included only 23 patients with some of them having had only benign tumors. Tumor sizes were small and could be resected totally. While in 17 patients (73.9%) negative margins could be achieved, six had contaminated margins; adjuvant radiotherapy was given to seven patients in that study. Supposedly, this was the reason why this study reported favorable treatment outcomes.

Due to Ewing’s sarcoma patients being the largest subgroup in this study, we also analyzed outcomes for Ewing’s sarcoma treatment separately. Therefore, local control and survival results are comparable to former series reporting 5-year OS of 50.3–73% and 5-year LC of 72–88% (7, 21–23). Only one report from Japan, in which multimodality treatment combined with proton therapy was used, showed substantially higher survival rate at 3 years of 92% and a high local control rate of 89.7% (24). Among all 35 non-metastatic Ewing’s sarcoma patients in the Japanese study, five had initially unresectable tumor of more than 8 cm and therefore had to receive higher radiotherapy dose. Whereas one of them received 59.4 Gy, the other four patients received even 64.8 Gy. None of the patients received doses in excess of 59.4 Gy encountered local relapsed of the disease. In our present study, the median total dose for Ewing sarcomas was 59.4 Gy, but in the majority of our cases the tumor was unresectable and had maximal diameter of more than 8 cm tumor. In addition, it included metastatic patients. This could explain the slightly superior treatment results of the Japanese study.

Regarding the outcome of osteosarcomas, our study resulted in high local control and survival rates with LC of 83.3% and both EFS and OS of 66.7% similarly at 2 years and 3 years even though this study had only one patient who underwent gross total surgery. These results are superior when compared to previous reports of pelvic osteosarcoma treated with photons (10, 25). When looking at previous proton therapy studies, findings are comparable (26). However, the number of patients with osteosarcoma in our study was limited and the median follow-up time for osteosarcomas was still limited with 16.4 months (9.3–55.6 months).

We also analyzed the factors potentially influencing on oncological outcome. Older patients have poor prognosis in all oncological outcomes. This negative impact of higher age has already been described in other studies indicating that younger patients have better survival after treatment particularly in Ewing’s sarcoma comprising the largest group in our analysis (5, 9, 16, 23). However, there is no definite cut-off level of age and a variety of cut-off levels were used in different studies. In our analysis, we used an age of 20 (young adult) as cut-off. However, we have to acknowledge that in patients of 20 years and more had a higher proportion of pelvic tumor sites (27), while younger cut-off level showed no significant impact on survival in the whole cohort patients. Besides higher age, also metastatic disease showed negative impact on survival in our study. This negative impact on survival outcome was observed across all sarcomas (28–31). Interestingly, also poor performance status was associated with decreased EFS. However, surgery did not show significant better local control or survival in our cohort which might because of insufficient number of patients in subgroup analysis. Among Ewing’s sarcoma patients alone, patients who were older than 20 years and tumor larger than 10 cm had worse prognosis for EFS. In accordance with our findings, the negative impact of older age and tumor size on survival has been revealed in other historical studies already (15, 16, 31). However, 8 cm of size which was used in many previous studies and TNM staging did not display any significant impact in our study. Radiotherapy given to the primary tumor for the first course of treatment showed better EFS in Ewing’s sarcoma patients when compared to patients who had radiotherapy at time of recurrence or when irradiated for metastatic sites which considered as higher risk disease. Surprisingly, none of the factors impacting on EFS appeared to affect overall survival. However, observation time may be too short to display the effect of the EFS on overall survival.

This study reported on 32 patients having relapsed. The most common pattern was isolated distant failure. However, local failure concerned more than half of all patients with or without dissemination. These results reflect the need for effective local therapy despite the high risk for dissemination for the majority of bone tumors. When highlighting the results of local PT, pattern of relapse with regard to high dose volume seems of particular importance. Our data suggest that the predominant site of failure was inside the high-dose region of PT. Only one patient experienced marginal failure but having synchronous widespread metastasis. Therefore, it is difficult to distinguish local marginal relapse from metastatic disease in this patient. This might be explained by the well-known radio-resistance of primary bone malignant tumors. It may be required to explore even higher dose or hypofractionation in the future to overcome of this radio-resistant nature. Presently, several studies are going to address dose escalation particularly in Ewing tumors with bulky residual disease (32).

Regarding toxicity profile, acute grades 3-4 toxicity were reported in 41 patients. The most common toxicity was hematological toxicity, which is not surprising as 69% of patients in this cohort received chemotherapy. All acute toxicity was manageable. Only three patients needed short-term hospitalization apart from receiving chemotherapy and only 2 patients had more than 3 days of PT interruption due to any toxicity-related. Furthermore, late toxicity of any higher grade was observed in only seven patients but not exceeding grade 3. Even though some data were missing, we still gathered about 82.7% of toxicity reports displaying a low rate of late toxicity so far. Two patients had undefined pain and bone pain, respectively. One patient treated osteosarcoma with total resection and adjuvant PT at age of 17 developed bone deformity, one patient had neuromotor problems, one patient had urinary incontinence and the last two patients had chronic diarrhea and fecal incontinence, respectively. Despite, one-third of patients received high dose radiotherapy of almost 70 Gy, low rate of late toxicity can be observed. This finding supports the idea of dose escalation of PT in this region can be well-tolerated.

It has to be acknowledged, that this analysis is limited due to its retrospective nature even if data collection within the registry was performed prospectively. Furthermore, selection bias cannot be excluded as the trial was not generated in a randomized fashion against other local therapies. Patients’ characteristics were also somewhat heterogeneous and difficult to compare because of the unbalanced nature of the data. In addition, some follow-up data were missing and could not be obtained. For surgical status, the recorded data did not distinguish between R0 or R1 status in some of patients who underwent gross total resection. Another limitation is the relatively small number of patients in our study particularly making statistical analyses difficult. We also have to acknowledge the limited follow-up periods. The median follow-up of this study was 27.5 months, and further investigation after longer follow-up will have to be done. Overall, longer follow-up and a greater number of patients is desirable to provide better evidence in the future.

## Conclusion

Multimodality treatment of pelvic and lumbar primary bone malignancy combined with proton therapy provided high local control and overall survival rates in a high-risk population despite limited extent of surgery for most of the patients. Isolated distant metastasis was the major cause of failure. However, local recurrence was occurring in more than half of the patients, predominantly situated within the high-dose radiotherapy region suggesting to further exploring dose escalation concepts for subcohorts of high-risk patients. Proton therapy was well feasible on a short term even when combined with chemotherapy and applied to typically large volumes. Therefore, not surprising, hematological toxicity was the most common acute toxicity followed by gastrointestinal toxicity. However, late toxicity has to be considered when applying locally intensive therapy. In our study, late complications were reported in less than 10% of our patients but after limited follow-up time and concerned predominantly musculoskeletal issues.

## Data Availability Statement

The original contributions presented in the study are included in the article/**Supplementary Material**. Further inquiries can be directed to the corresponding author.

## Ethics Statement

The studies involving human participants were reviewed and approved by the Ethik-Kommission der Medizinischen Fakultät der Universität Duisburg-Essen. Written informed consent to participate in this study was provided by the participants’ legal guardian/next of kin.

## Author Contributions

RW, TS, and BT contributed to conception and design of the study. RW, TS organized the database. RW performed the statistical analysis. RW wrote the first draft of the manuscript. RW, TS and BT edited the final manuscript. All authors listed have made a substantial, direct, and intellectual contribution to the work and approved it for publication.

## Conflict of Interest

The authors declare that the research was conducted in the absence of any commercial or financial relationships that could be construed as a potential conflict of interest.

## Publisher’s Note

All claims expressed in this article are solely those of the authors and do not necessarily represent those of their affiliated organizations, or those of the publisher, the editors and the reviewers. Any product that may be evaluated in this article, or claim that may be made by its manufacturer, is not guaranteed or endorsed by the publisher.
