# Supplementary material for: Proton Therapy for Primary Bone Malignancy of the Pelvic and Lumbar Region – Data From the Prospective Registries ProReg and KiProReg
Source: Front Oncol. 2022 Feb 16;12:805051. doi: 10.3389/fonc.2022.805051 (PMC8888414; doi:10.3389/fonc.2022.805051)

Supplementary 1: CTV delineation according to histology.

| Histology | CTV1 | CTV2 | CTV3 |
| --- | --- | --- | --- |
| Ewing’s sarcoma | Tumor bed + 1.5-2 cm | Tumor bed | GTV2** |
| Chordoma | Tumor bed + 1 cm | GTV2 | - |
| Chondrosarcoma | Tumor bed + 1 cm | GTV2 | - |
| Osteosarcoma | Tumor bed + 2 cm | GTV2 | - |
| Osteoblastoma | Tumor bed + 0.5-2 cm | - | - |

** Boost can be considered if residual still present at the start of radiotherapy

Supplementary 2: Demographic data and radiotherapy parameters

|  | Ewing’s sarcoma | Chordoma | Chondrosarcoma | Osteosarcoma | Osteoblastoma |
| --- | --- | --- | --- | --- | --- |
| Number of patients (%) | 47 (58) | 20 (24.7) | 6 (7.4) | 6 (7.4) | 2 (2.5) |
| Mean age at diagnosis  (range) | 16.37  (1.33 - 42.92) | 59.32  (12.33 – 85.75) | 54.53  (35.67 – 69.25) | 26.83  (10.08 – 66.67) | 12.67  (12.25 -13.08) |
| Sex  Female  Male | 23  24 | 13  17 | 2  4 | 1  5 | 0  2 |
| ECOG performance status  0-1  2  3  4  Unknown | 35  7  5  0  - | 14  1  2  1  2 | 4  0  2  0  - | 3  1  2  0  - | 2  0  0  0  - |
| Location of tumor  Pelvic and sacrum  Lumbar | 39  8 | 18  2 | 5  1 | 5  1 | 1  1 |
| Tumor size (median in cm)  (range) | 8.05  (2.80 – 20.00) | 6.40  (1.50 – 16.00) | 10.15  (4.80 – 20.00) | 11.88  (8.00 – 17.60) | 4.70  (1.40 – 8.00) |
| N staging  N0  N1 | 45  2 | 20  0 | 6  0 | 6  0 | 2  0 |
| M staging  M0  Lung only  Non-Lung  Combined | 38  4  1  4 | 19  0  1  0 | 5  1  0  0 | 2  2  0  2 | 2  0  0  0 |
| Indication of PT  Definite  Adjuvant  Pre-operative | 30  11  6 | 15  5  0 | 5  1  0 | 5  1  0 | 2  0  0 |
| Median radiation Dose  (range)  Definite  Adjuvant  Pre-operative | 59.4  (45 – 59.4)  59.4 (50.4 – 59.4)  54 (45 – 59.4)  50.4 (45 – 54) | 73.5  (70 – 74)  73.5 (72 – 74)  73.5 (73.5 – 74) | 69.3  (68 – 73.5)  69.3 (69.3 -73.5)  68 (68) | 70  (56 – 70)  70 (69.8 – 70)  56 (56) | 54  (54 – 54)  54 (54 – 54) |
| Technique of PT  Sequential  SIB | 47  0 | 10  10 | 2  4 | 5  1 | 2  0 |

Abbreviations:

PT = Proton therapy

SIB = Simultaneous integrated boost

Supplementary 3: Univariate analysis of all patients

| Factors (N) | Local control | | EFS | | OS | |
| --- | --- | --- | --- | --- | --- | --- |
|  | 2-year LC (%) | p value | 2-year EFS (%) | p value | 2-year OS (%) | p value |
| Age  ≤ 20 years (42)  > 20 years (39) | 91.1  62.3 | <0.01 | 76.6  44.6 | <0.01 | 93.9  82.2 | 0.10 |
| Sex  Female (29)  Male (52) | 73  78.3 | 0.83 | 49.1  65.2 | 0.48 | 91.2  86.4 | 0.11 |
| Performance status  0-1 (58)  2-4 (21) | 77.7  70.7 | 0.32 | 62.2  46.5 | 0.09 | 90.1  80.5 | 0.13 |
| Location  Pelvic/sacrum (68)  Lumbar (13) | 76.8  73.8 | 0.86 | 61.6  51.3 | 0.72 | 87.2  92.3 | 0.39 |
| Histology  Ewing’s sarcoma (47)  Chordoma (20)  Chondrosarcoma (6)  Osteosarcoma (6)  Osteoblastoma (2) | 80.2  73.2  50  83.3  100 | 0.19 | 61.7  67.8  16.7  66.7  100 | 0.13 | 88.7  95  66.7  66.7  100 | 0.51 |
| Tumor size  ≤ 10 cm (62)  > 10 cm (18) | 79.5  62.5 | 0.24 | 66.1  39.3 | 0.08 | 88.4  88.9 | 0.2 |
| N staging  N0 (79)  N+ (2) | 77.5  - | 0.26 | 60.7  - | 0.53 | 87.9  100 | 0.09 |
| M staging  M0 (66)  M+ (15) | 72.9  93.3 | 0.15 | 60.2  57.9 | 0.73 | 89.1  82.5 | 0.04 |
| Surgery  No resection (41)  Any resection (37) | 72.4  79.2 | 0.27 | 50.5  68.7 | 0.06 | 86.6  88.5 | 0.69 |
| Chemotherapy  No (25)  yes (56) | 70.5  79.1 | 0.14 | 66.2  56.1 | 0.82 | 91.6  86.2 | 0.31 |
| PT indication  Definite (57)  Adjuvant (18)  Pre-operative (6) | 75.8  77.9  80 | 0.89 | 55.7  69.2  80 | 0.47 | 85.8  91.7  100 | 0.65 |
| PT technique  Sequential (66)  SIB (15) | 77.9  72.7 | 0.32 | 60.1  62.3 | 0.76 | 89.1  85.1 | 0.83 |
| Site of PT  Primary (70)  Recurrence (7)  Primary with metastatic site (4) | 74.4  85.7  100 | 0.6 | 62.4  34.3  75 | 0.36 | 88.1  100  66.7 | 0.16 |
| Response to chemotherapy  ≥ 66% (36)  < 66% or PD (45) | 82.3  72.4 | 0.19 | 71.6  52 | 0.11 | 92.3  84.8 | 0.23 |
| Scar  Whole scar not covered by PT (57)  PT covered whole scar (24) | 74.6  80.7 | 0.82 | 55.9  69.9 | 0.21 | 84.9  95.5 | 0.2 |

( - ) = Last patient at risk was censored before 2 years

Supplementary 4: Univariate analysis of Ewing’s sarcoma patients

| Factors (N) | Local control | | EFS | | OS | |
| --- | --- | --- | --- | --- | --- | --- |
|  | 2-year LC (%) | p value | 2-year EFS (%) | p value | 2-year OS (%) | p value |
| Age  ≤ 20 years (36)  > 20 years (11) | **89.3**  **56.3** | **0.06** | **75**  **30.3** | **<0.01** | 92.7  78.8 | 0.17 |
| Sex  Female (23)  Male (24) | 82.4  79.1 | 0.98 | 57.2  65.2 | 0.92 | 93.8  83.9 | 0.44 |
| Performance status  0-1 (35)  2-4 (12) | 78.3  87.5 | 0.67 | 58.9  72.9 | 0.73 | 89.7  85.7 | 0.74 |
| Location  Pelvic/sacrum (39)  Lumbar (8) | 79.5  88.3 | 0.94 | 62.4  58.3 | 0.82 | 89  87.5 | 0.98 |
| Tumor size  ≤ 10 cm (36)  > 10 cm (10) | **85.4**  **57.1** | **0.02** | **71.7**  **25.7** | **0.05** | 88.7  90 | 0.24 |
| N staging  N0 (45)  N1 (2) | 82.2  - | 0.57 | 63  - | 0.34 | **88.3**  **100** | **0.05** |
| M staging  M0 (38)  M1 (9) | 75.4  100 | 0.16 | 62.3  57.1 | 0.64 | 89.2  85.7 | 0.28 |
| No resection (24)  Any resection (23) | 78.9  79.4 | 0.27 | 54.7  66.5 | 0.34 | 95.2  79 | 0.63 |
| PT indication  Definite (30)  Adjuvant (11)  Pre-operative (6) | 77.5  88.9  - | 0.82 | 54.3  76.2  80 | 0.56 | 87  87.5  100 | 0.94 |
| Site of PT  Primary (42)  Recurrence or Primary with  metastatic site (5) | 77.5  100 | 0.59 | **68.8**  **0** | **0.01** | **90.2**  **80** | **0.07** |
| Volume response from chemotherapy (calculated from planning system)  ≥ 66% (28)  < 66% or PD (19) | 87  70.6 | 0.39 | 73.1  46.2 | 0.31 | 90.4  85.6 | 0.54 |

( - ) = Last patient at risk was censored before 2 years

Supplementary 5: Multivariate analysis of Ewing’s sarcoma patients

| Factors | Local control | | EFS | | OS | |
| --- | --- | --- | --- | --- | --- | --- |
|  | HR (95% CI) | p value | HR (95% CI) | p value | HR (95% CI) | p value |
| Age  ≤ 20 vs > 20 | 4.14  (0.74 – 23.17) | 0.11 | **4.51**  **(1.65-12.29)** | **<0.01** | 3.86  (0.67 – 22.39) | 0.13 |
| Tumor size  ≤ 10 vs > 10 cm | 2.41  (0.43 – 13.63) | 0.32 | **4.16**  **(1.29 – 13.48)** | **0.02** | 1.87  (0.28 – 12.53) | 0.52 |
| M staging  M0 vs M1 | 0 | 0.97 | 0.97  (0.19 – 4.88) | 0.97 | 2.35  (0.31 – 18.12) | 0.41 |
| Site of PT  Primary vs Recurrence/Primary with metastatic site | 0 | 0.98 | **7.59**  **(2.01 – 28.71)** | **<0.01** | 3.88  (0.48 – 31.21) | 0.2 |

Supplementary 6: Kaplan–Meier estimates of event-free survival rates for all patients of this study according to age (A), performance status (B), tumor size (C) and resection status (D).


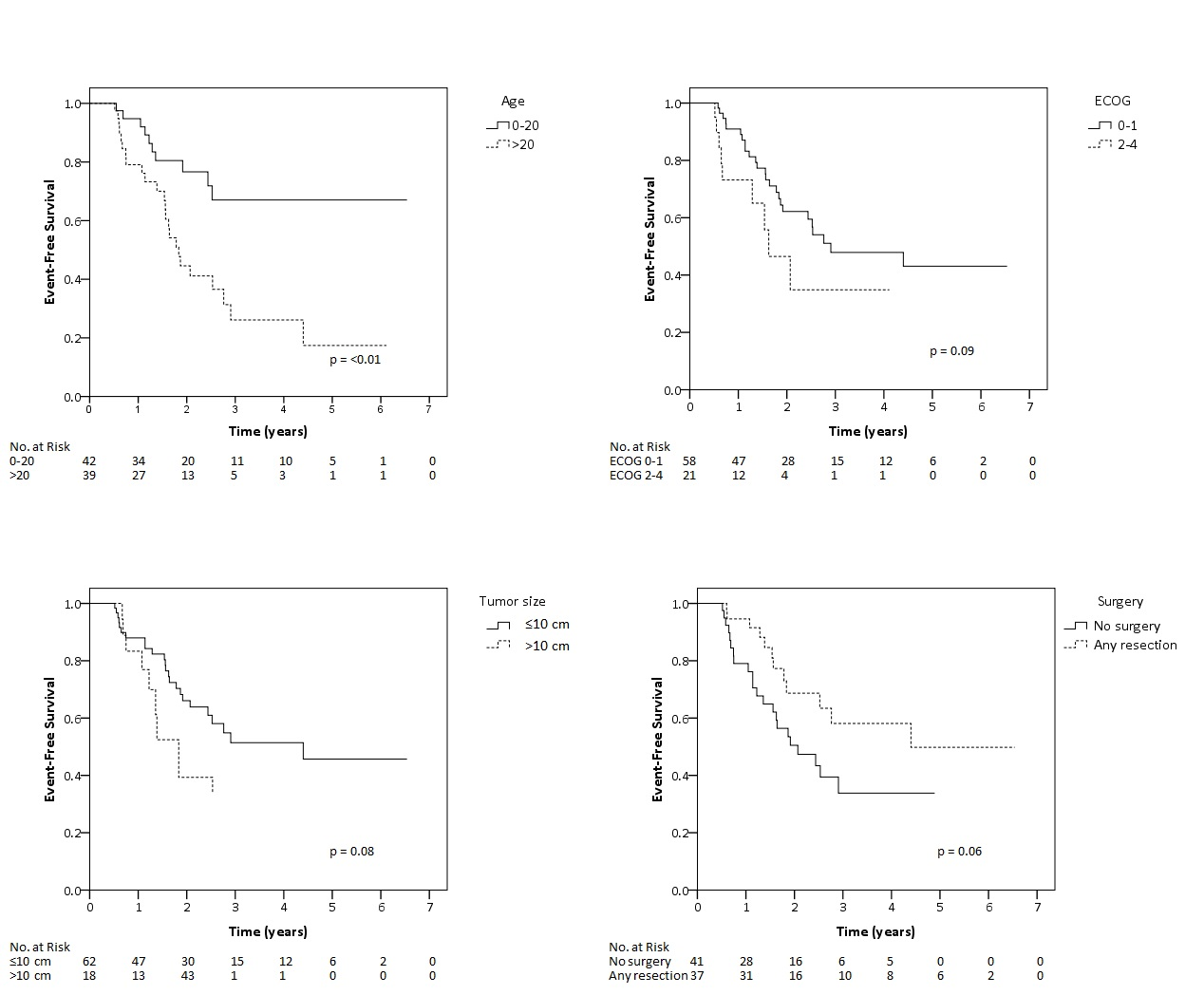

Supplement: Supplementary file 1 [file DataSheet_1.docx]
